# Low-carbon footprint diluents in solvent extraction for lithium-ion battery recycling†

**DOI:** 10.1039/d3ra04679f

**Published:** 2023-08-02

**Authors:** Aboudaye M. Ahamed, Benjamin Swoboda, Zubin Arora, Jean Yves Lansot, Alexandre Chagnes

**Affiliations:** a Université de Lorraine, CNRS, GeoRessources F-54000 Nancy France alexandre.chagnes@univ-lorraine.fr; b TotalEnergy Fluids 2 Place Jean Miller, La Défense Cedex 92078 Paris France

## Abstract

This study investigated the influence of the diluent on the extraction properties of three extractants towards cobalt(ii), nickel(ii), manganese(ii), copper(ii), and lithium(i), *i.e.* Cyanex® 272 (bis-(2,4,4-trimethylpentyl)phosphinic acid), DEHPA (bis-(2-ethyl hexyl)phosphoric acid), and Acorga® M5640 (alkylsalicylaldehyde oxime). The diluents used in the formulation of the extraction solvents are (i) low-odour aliphatic kerosene produced from the petroleum industry (ELIXORE 180, ELIXORE 230, ELIXORE 205 and ISANE IP 175) and (ii) bio-sourced aliphatic diluents (DEV 2138, DEV 2139, DEV 1763, DEV 2160, DEV 2161 and DEV 2063). No influence of the diluent and no co-extraction of lithium(i), nickel(ii), cobalt(ii), manganese(ii) and aluminum were observed during copper(ii) extraction by Acorga M5640. The nature of the diluent influenced more significantly the extraction properties of manganese(ii) by DEHPA as well as cobalt(ii) and nickel(ii) by Cyanex® 272. Life cycle assessment of the diluents shows that the carbon footprints of the investigated diluents followed the following order: (ELIXORE 180, ELIXORE 230, ELIXORE 205) from petroleum industry > kerosene from petroleum industry > diluent produced from tall oil (DEV 2063) > diluents produced from recycled plastic (DEV 2160, DEV 2161) > diluents produced from used cooking oil (DEV 2138, DEV 2139). By taking into account the physicochemical properties of these diluents (viscosity, flashpoint, aromatic content), the extraction properties of Acorga® M5640, DEHPA, Cyanex® 272 in these diluents and the CO_2_ footprint of the diluents, this study showed DEV2063 and DEV2139 were the best diluents. A low-carbon footprint solvent extraction flowsheet using these diluents was proposed to extract selectively cobalt, nickel, manganese, lithium and copper from NMC black mass of spent lithium-ion batteries.

## Abstract 2

This study investigated the influence of low-carbon footprint diluents and diluents from petroleum industry on Co(ii), Ni(ii), Mn(ii), Li(i) and Cu(ii) extraction by Cyanex® 272, DEHPA and Acorga® M5640.

## Graphical abstract

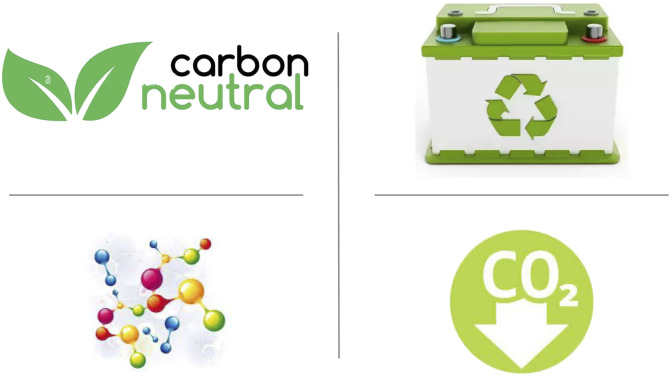

## 1. Introduction

Population growth and rapidly evolving technologies are triggering a strong demand for metals such as lithium, nickel, cobalt, manganese and copper. These metals are most used in lithium battery manufacturing (71%).^1^ Lithium-ion batteries (LiBs) offer many advantages, most notably their ability to store relatively large amounts of energy compared to other systems, their high charging capacity and their long-life span.^2^ For this reason, lithium-ion batteries are considered the best technology for electric vehicles and stationary applications, and an exponential growth of the global demand of lithium, nickel, cobalt and manganese is expected in the coming years.^3,4^ The circular economy stimulates the development of sustainable and efficient recycling technologies to mitigate the environmental impact of lithium-ion battery production and to improve resource management.

In practice, spent LiBs are dismantled after a deep discharge, shredded to liberate the different components of the batteries, and valuable elements (usually nickel, cobalt, manganese, lithium, copper, aluminum, and graphite) are concentrated in a blackmass by using different physical separation methods such as froth flotation, magnetic separation, eddy current, screening, gravimetric separation, *etc.* This blackmass undergoes hydrometallurgical operations, *i.e.* leaching, solvent extraction, precipitation, *etc.*^5^ Leaching, is usually carried out in sulfuric acid in the presence of hydrogen peroxide^6,7^ but other studies showed that hydrochloric acid could be alternatively used.^8,9^

Metal extraction and metal separation are key steps of recycling processes to produce high-grade salts. These steps can be performed by liquid–liquid extraction, liquid–solid extraction, precipitation–crystallization, and (electro)-membrane processes.^10–14^ Among these technologies, liquid–liquid extraction is widely used to extract, concentrate and separate metals from the leach solution.^15,16^ This technique consists of mixing the leach solution with a non-miscible extraction solvent containing an extractant and a diluent in order to extract selectively the target metal(s). The performance of the liquid–liquid operation depends on the choice of the extractant and the diluent beside the use of optimized contactors (mixer-settler or column) under optimized conditions (pH, temperature, flowrates, *etc.*). The diluent is usually an aliphatic diluent which exhibits high chemical stability. The use of different extractants (DEHPA, Cyanex® 272, TOPO, PC-88A, *etc.*) and various diluents containing more or less aromatic compounds are reported in literature for the efficient and selective recovery of lithium, cobalt, nickel, manganese and copper (Table 1). Other free-diluent systems are reported in literature to replace conventional extraction solvents such as ionic liquids or deep eutectic solvents.^17,18^ However, there is no study reported in literature regarding the impact of the use of these systems on the CO_2_ emission of the solvent extraction process. Darvishi *et al.*^19^ reported that DEHPA does not exhibit a good selectivity for cobalt–nickel separation at any concentrations and temperatures since the differences in pH of half extraction between nickel and cobalt (pH_1/2,Ni_–pH_1/2,Co_) are equal to 0.26, 0.58 and 0.59 at 25, 40 and 60 °C, respectively.

**Table 1:** Liquid–liquid extraction systems reported in the literature to extract nickel(ii), cobalt(ii) and manganese(ii) from acidic aqueous media

Objectives	Aqueous phase composition (g L^−1^)	Diluent	Extractant	Ref.
Direct production of Ni–Co–Mn mixtures for cathode precursors from cobalt-rich LiBs leachates by solvent extraction	Li = 2.5	Exxsol D80	D2EHPA	41
	Ni = 2			
	Co = 16.8			
	Mn = 2.1			
Selective recovery of cobalt, nickel and lithium from sulfate leachate of cathode scrap of LiBs using liquid–liquid extraction	Co = 25.1	Kerosene	PC-88A	51
	Ni = 2.54			
	Li = 2.62			
Metal separation from mixed types of batteries using selective precipitation and liquid–liquid extraction techniques	Co = 0.37	Kerosene	Cyanex® 272	52
	Ni = 1.24			
	Cu = 0.301			
	Mn = 0.499			
Mechanical and hydrometallurgical processes in HCl media for the recycling of valuable metals from LiB waste	Ni = 1.295	Kerosene	Cyanex® 272	53
	Co = 6.976			
	Ni = 0.821			
	Mn = 0.992			
	Cu = 0.113			
Hydrometallurgical process for recovery of cobalt from waste cathodic active material generated during manufacturing of LiBs	Co = 44.72	Kerosene	Cyanex® 272	54
	Li = 5.43			
Separation of cobalt and lithium from mixed sulphate solution using Na–Cyanex® 272	Co = 0.01	Kerosene	Na–Cyanex® 272	55
	Li = 0.01			
Selective extraction and separation of metal values from leach liquor of mixed spent LiBs	Co = 32.7	Kerosene	Na–Cyanex® 272	56
	Li = 2.26			
	Ni = 0.07			
	Mn = 18.6			
Development of a novel solvent extraction process to recover cobalt, nickel, manganese, and lithium from cathodic materials of spent LiBs	Li = 1.441	Kerosene	Alamine® 336	8
	Co = 3.67			
	Ni = 3.60			
	Mn = 3.61			
Hydrometallurgical recovery of metal values from sulfuric acid leaching liquor of spent LiBs	Ni = 6.41	Kerosene	DEHPA	6
	Co = 6.09			
	Mn = 6.29			
	Li = 1.60			
Recovery of metals from spent LiBs leach solutions with a mixed solvent extractant system	Co = 16.9	Shellsol D70	Ionquest® 801 + Acorga® M5640	57
	Li = 3.8			
	Ni = 0.15			
Improvement of metal separation process from synthetic hydrochloric acid leaching solution of spent LiBs by solvent extraction and ion exchange	Cu = 150	Kerosene	Cyanex® 301	58
	Co = 0.938			
	Mn = 0.15			
	Ni = 0.1			
	Li = 0.15			
Lithium recovery from effluent of spent LiB recycling process using solvent extraction	Li = 1.85	Kerosene	HBTA and TOPO	59
	Co = 0.005			
	Ni = 0.025			
The separation and recovery of nickel and lithium from the sulfate leach liquor of spent LiBs using PC-88A	Li = 4.82	Kerosene	PC-88A	7
	Ni = 2.54			

Conversely, Devi *et al.*^20^ demonstrated that Cyanex® 272 is the best extractant compared to DEHPA and PC-88A to separate cobalt(ii) and nickel(ii). Tait *et al.* investigated the separation of cobalt(ii) and nickel(ii) by using Cyanex® 272, Cyanex® 301 and Cyanex® 302 diluted in toluene.^21^ They showed that Cyanex® 272 can separate cobalt(ii) and nickel(ii) very efficiently as the pH values of half extraction for cobalt(ii) are equal to 1.3, 4.0 and 5.3 whereas the pH values of half extraction for nickel are pH_1/2,Ni_ = 2.4, 6.6 and 7.0 when 0.25 mol per L Cyanex® 301, Cyanex® 302 or Cyanex® 272 are used as extractant, respectively. These results agree with those obtained by Sole and Hiskey.^22^

Cobalt(ii)–manganese(ii) separation was also studied by several authors. Devi *et al.* investigated cobalt(ii)–manganese(ii) separation in acidic sulfate media by using Cyanex® 272, DEHPA and PC-88A.^23^ They showed manganese(ii) and cobalt(ii) can be separated efficiently from acidic sulfate solution at pH 2.7 by using DEHPA. With respect to the efficiency of separation, these authors showed that DEHPA was better than PC88A and Cyanex® 272. Others authors^24^ showed that the separation was even better by controlling the pH from 2 to 4 when DEHPA was used as extractant.

The physical properties of the diluent, *i.e.* density, viscosity, dielectric constant and solubility parameters, influence the extraction of the metal from the leach solution into the organic phase.^25^ In general, aromatic diluents have higher densities than aliphatic diluents, which may impede the dispersion and coalescence whereas the polarity of the diluent has a huge effect on the extraction efficiency. Furthermore, the interactions in solution may be responsible for self-association of the extractants. The intensity of self-association depends on the polarity of the diluent : extractant molecules tend to form monomer in polar diluents and dimers, trimers or even oligomers in non-polar diluents.^26,27^ The average degree of polymerisation increases with a decrease in polarity of the diluent. The presence of polymeric forms decreases the loading capacity of the extractant whereas the interaction of the diluent with the extractant can decrease the extraction efficiency since the formation of extractant–diluent species results in a lower concentration of free extractants. Furthermore, diluents affect the solvation of the extractant and, hence, its extractive properties. There are only few studies regarding the effect of the nature of the diluent on the extraction performance of liquid–liquid extraction, but no study concerns the effect of diluents on cobalt(ii)–nickel(ii)–manganese(ii) separation for LiBs recycling processes. In many cases, the distribution ratios cannot be correlated to the physical properties of the diluent, even though many attempts have been made to do so. Taube tried to correlate the extraction efficiency of uranium, plutonium and neptunium with the dipole moment and the dielectric constant of the solvent.^28,29^ Healy^30^ linked the diluent effect with the water content of the solvents. The effect of diluents on the solvent extraction of metal ions has been studied for copper(ii),^31–34^ cobalt(ii),^35^ nickel(ii),^36–38^ uranium(vi).^39^

It is of great importance to take into consideration the diluent during the formulation of an extraction solvent since (i) the diluent may have a significant effect on the extraction properties of a solvent in liquid–liquid extraction, and (ii) the diluent, which is the main component of an extraction solvent, impacts directly the environmental footprint of the liquid–liquid extraction process. Furthermore, the European Regulation (EC) No. 1907/2006 of 18/12/06 (ref. 40) concerning the Registration, Evaluation, Authorization and Restriction of Chemicals encourages the development of industrial processes that use less toxic organic compounds. It is therefore necessary to use eco-friendly reagents in hydrometallurgical processes. In the case of liquid–liquid extraction, bio-sourced diluents may be an alternative to petroleum kerosene to reduce the CO_2_ footprint of the liquid–liquid extraction operation.

In this paper, we compared the extraction properties of different extraction solvents composed of extractants (Acorga M5640, DEHPA, Cyanex® 272) in petroleum diluents (ELIXORE180, ELIXORE230, ELIXORE 205 and ISANE IP175) and in bio-sourced aliphatic diluents (DEV 2138, DEV2139, DEV1763, DEV2160, DEV2161 and DEV2063) for the recovery of lithium(i), nickel(ii), cobalt(ii), manganese(ii) and copper(ii) from acidic sulfate media representative of a leaching solution produced by dissolving a blackmass of spent lithium-ion battery in sulfuric acid. The best extraction solvent has been selected from the extraction performance and the CO_2_ footprint of the diluents. Then, a flowsheet has been proposed to extract and separate the different metals contained in the leach solution by using the selected diluents.

## 2. Experimental

### 2.1. Chemical reagents

Aqueous solutions were prepared by dissolving copper sulfate (CuSO_4_·5H_2_O, Aldrich, purity ≥ 99%), lithium sulfate (Li_2_SO_4_,H_2_O, Aldrich, purity ≥ 99.0%), cobalt sulphate (CoSO_4_·7H_2_O, Aldrich, purity ≥ 99%), manganese sulphate (MnSO_4_·H_2_O, Aldrich, purity ≥ 99%), nickel sulfate (NiSO_4_·6H_2_O, Aldrich, ≥ 98%) and aluminum(iii) sulfate (Al_2_(SO_4_)_3_, 14H_2_O, Aldrich, purity = 98%) in 0.35 mol per L sulfuric acid. The solution of 0.35 mol per L sulfuric acid was prepared by diluting an appropriate amount of concentrated sulfuric acid (Aldrich, 95%) in deionized water (resistivity > 18 MΩ cm).

Table 2 shows the composition of the aqueous solution representative of a leaching solution produced by dissolving NMC cathode materials with sulfuric acid in the presence of hydrogen peroxide.^41^

**Table 2:** Composition of the representative leach solution

Element	Li	Ni	Co	Mn	Al	Cu
Concentration (g L^−1^)	2.7	2.6	14.4	2.7	1.2	1.6

A solution of 10 mol per L sodium hydroxide was prepared by dissolving the appropriate amount of sodium hydroxide pellets (NaOH, Aldrich, purity = 99%) in deionized water (resistivity > 18 MΩ cm). This solution was used to adjust the pH of the aqueous solution during the solvent extraction experiments. The pH values of the aqueous solution before and after extraction were measured using a 1000 L pH meter (VWR) equipped with a pH electrode phenomenal 221 (VWR).

The organic phases for the liquid–liquid experiments were prepared by diluting the appropriate amounts of the extractants in the diluents. The following extractants used in the present work were kindly provided by Solvay: Cyanex® 272 (bis-(2,4,4-trimethylpentyl)phosphinic acid, purity = 90%), DEHPA (bis(2-ethylhexyl)phosphoric acid, purity = 95%) and Acorga® M5640 (5-nonyl-2-hydroxy-benzaldoxime, purity not specified). The diluents reported in Table 3 were kindly provided by TotalEnergy, except the petroleum aliphatic kerosene which was supplied by Aldrich (low-odour kerosene). It is interesting to point out that the bio-sourced diluents derived from waste oil, recycled plastic or vegetable oil exhibit higher flash points than petroleum-based diluents and they contain fewer aromatics, but they are slightly more viscous than diluents from petroleum industry.

**Table 3:** Flash point (FP), aromatic content and kinematic viscosity at 40 °C (*υ*) of the investigated diluents (IP: isoparaffine, LP: linear paraffine, A: aromatics, N: naphtene)

	Category	FP (°C)	Aromatic (ppm)	*υ* at 40 °C (mm^2^ s^−1^)	Composition (%)			
					IP	LP	A	N
Kerosene	Fossil	52	—	1.1	20	10	20	50
EXILORE180	Fossil	66	<300 ppm	1.4	20	10	0	70
EXILORE230	Fossil	104	<300 ppm	2.4	30	10	0	60
EXILORE205	Fossil	75	<300 ppm	1.7	20	10	0	70
ISANE IP175	Fossil	63	<50 ppm	1.23	95	5	0	0
DEV2138	Used cooking oil	64	<50 ppm	1.17	80	20	0	0
DEV2139	Used cooking oil	84	<50 ppm	1.61	80	20	0	0
DEV1763	Vegetable oil	115	<50 ppm	2.37	95	5	0	0
DEV2160	Recycled plastic	49	<100 ppm	1.07	35	35	0	30
DEV2161	Recycled plastic	92	<100 ppm	1.88	30	40	0	30
DEV2063	Tall oil	61	<50 ppm	1.23	45	10	0	45

### 2.2. Solvent extraction

Solvent extraction experiments were performed by contacting 10 mL of the aqueous phase with 10 mL of the organic phase in a centrifuge tube (50 mL). The pH of the aqueous phase was adjusted with 10 mol per L NaOH. No precipitation was observed during liquid–liquid extraction experiments (the solution was clear and no solid was formed at the bottom of the flask and at the liquid–liquid interface). The two non-miscible phases were mixed during 15 minutes at room temperature and 200 rpm with a mechanical stirring apparatus (Gerhardt Laboshake) thermostated with a Gerhardt Thermoshake, so that the equilibrium was reached. A mixing time of 15 minutes is enough since literature data showed that the steady state with the investigated extractants can be reach after contacting the aqueous and organic phases during less than 5 minutes.^42,43^ Samples were centrifuged at 3000 rpm for 2 min by means of a Sigma 3-16L Compact Benchtop Centrifuge in order to separate the organic and the aqueous phases. Before analysing the metal concentrations in the aqueous phases, the aqueous solutions were filtered to remove traces of organic phase with a hydrophilic filter (Minisart NML 16555K, cellulose acetate, 0.45 μm, *d* = 28 mm, VWR).

Elemental analyses were performed using a microwave plasma-atomic emission spectrometer (MP-AES 4210, Agilent). The wavelengths used for elemental analyses of lithium, nickel, cobalt, manganese, copper and aluminum were 497.175 nm, 361.939 nm, 350.631 nm, 294.920 nm, 324.754 nm and 308.215, respectively. The samples were diluted in 2% (vol) nitric acid prepared from a concentrated solution (HNO_3_, 95%, Aldrich) by dilution in deionized water (resistivity > 18 MΩ cm). Standards for MP analyses were prepared by diluting commercial standards provided by VWR containing 1000 ppm Li, Co, Mn, Ni, Al and Cu in 2% (vol) HNO_3_. Standard compositions were adjusted by adding sulfuric acid (0.35 mol L^−1^) in order to reach the same sulfate concentration as in the sample to analyse, and therefore, to reduce interference phenomena during MP analyses. The metal concentrations in the aqueous solutions before and after solvent extraction were used to calculate the extraction efficiency of the metal M (M = Li, Ni, Mn, Co, Cu or Al) by using the following equation:1
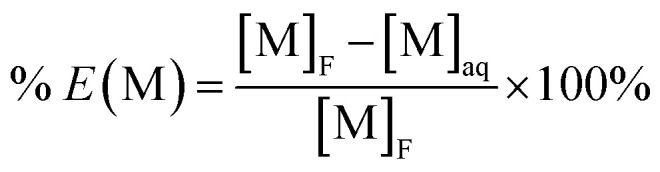
where [M]_F_ and [M]_aq_ denote the initial concentration of the metal M in the feed solution and the metal concentration in the aqueous phase in contact with the organic phase at the equilibrium at a phase volume ratio O/A = 1, respectively.

## 3. Results and discussion

### 3.1. Influence of diluents on the extraction curves

**Fig. 1:**
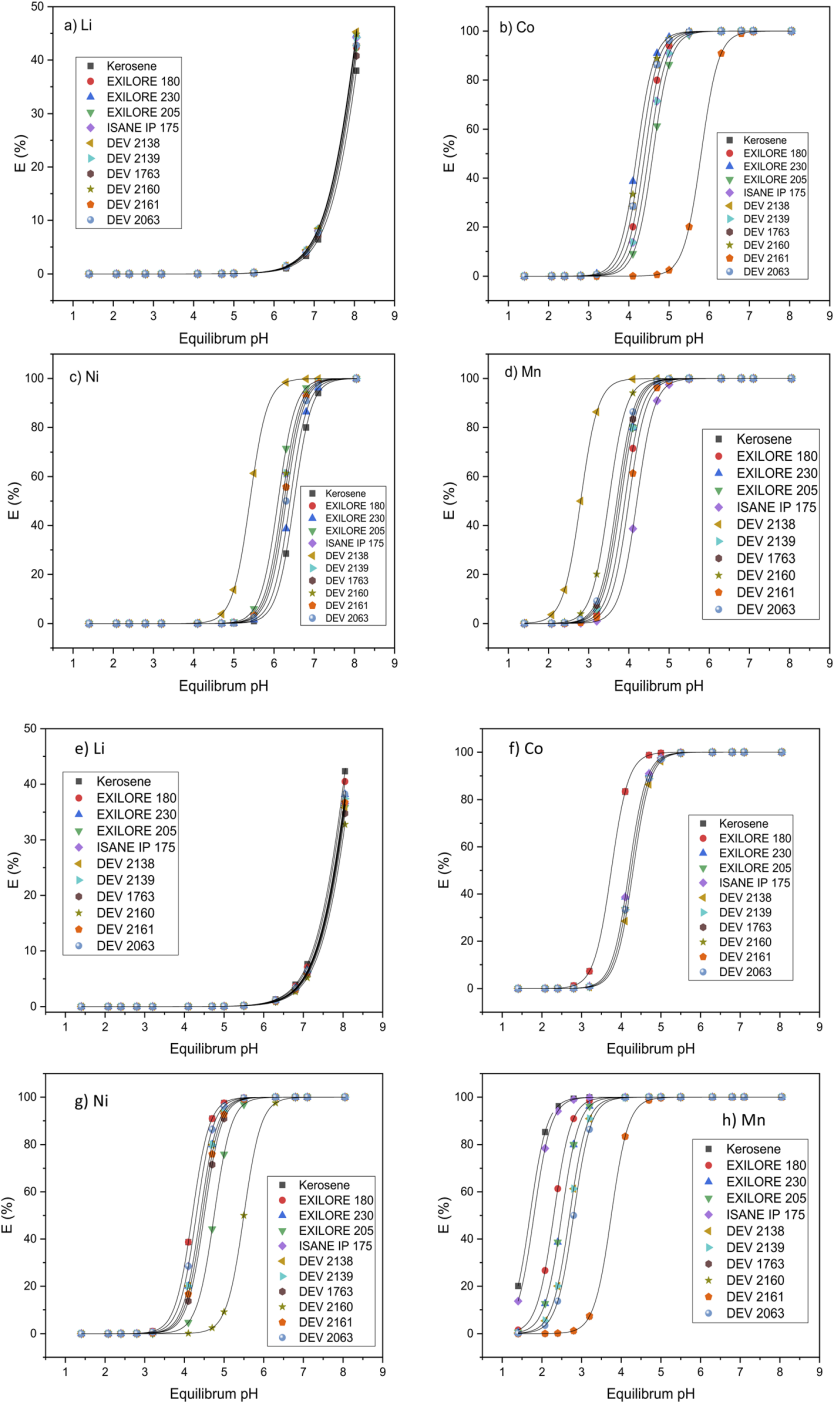
Influence of the diluents on the extraction efficiency of lithium(i), cobalt(ii), nickel(ii) and manganese(ii) by (a)–(d) 1 mol L^−1^ Cyanex® 272 and (e)–(h) 0.8 mol L^−1^ DEHPA as a function of pH (O/A = 1, room temperature).

The influence of the diluents reported in Table 3 on the extraction efficiency by DEHPA and Cyanex® 272 of cobalt(ii), nickel(ii), manganese(ii) and lithium(i) diluted in the acidic aqueous solution representative of leach solution (see composition in Table 2) is reported in Fig. 1. In the present study, the influence of the diluent and the extractant on the extraction efficiency of aluminum has not been studied as aluminum is usually precipitated before liquid–liquid extraction (see precipitated curves in Fig. S1, ESI†). Therefore, the composition of the aqueous solutions used in the present work contains all metals at concentrations reported in Table 2 except aluminum as it is inferred that aluminum was removed by precipitation before liquid–liquid extraction. The diluent influences the extraction properties of these metals except for copper and lithium (see below). The general rule for the extraction effect of the diluent on the extraction is that a higher dielectric constant and a higher dipole moment lead to an increase in extraction efficiency.^44–46^ This is explained by the fact that the interactions between the extractant molecules and the diluent molecules are stronger if the dielectric constant of the diluent is higher. These stronger interactions result in a weaker extraction of metal ions. However, in the present study, it is expected that the dielectric constant of the different diluents reported in Table 3 are similar and close to *ε*_r_ = 2–4. Therefore, the difference in extraction behavior observed for several systems cannot be explained by dielectric constants.

Liquid–liquid extraction of cobalt(ii), nickel(ii) and manganese(ii) from acidic aqueous media by cationic exchangers such as DEHPA, Cyanex® 272 and Acorga® M5640 involves the following general equilibrium:^47–50^2
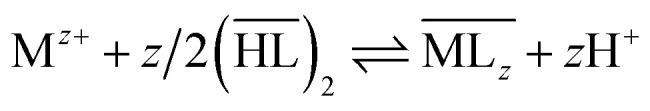
where M^*z*+^ represents Co(ii), Ni(ii), Mn(ii) or Cu(ii), and HL denotes the cationic exchanger (DEHPA, Cyanex® 272, Acorga® M5640). The overbar indicates that the species are in the organic phase whereas the absence of overbar indicates the species are in the aqueous phase (for a sake of simplification, this equation does reflect the potential involvement of HL molecules in the solvation shell of the extracted species ML_*z*_(HL)_*x*_).

The following equation can be deduced from eqn (2) to fit the experimental curves of the extraction efficiency as a function of pH:3
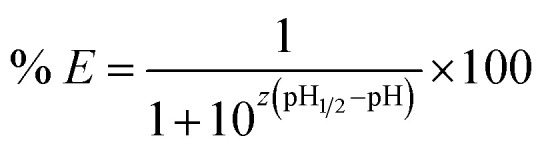
where pH_1/2_ is the pH value for which the extraction efficiency is equal to 50% (half-extraction pH).

The extraction constant (*K*_ex_) of the extraction reaction (2) is defined as:4
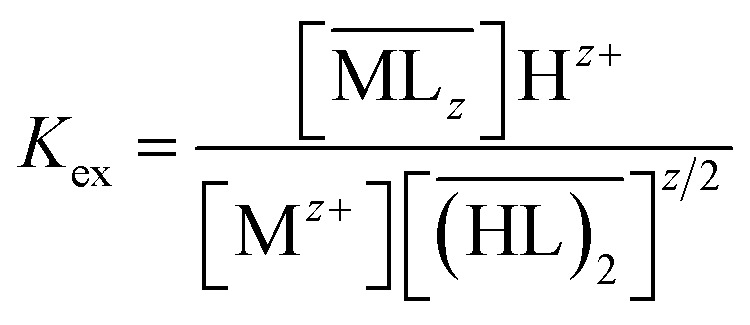


The following equilibrium constants can be used to rewrite eqn (4):5
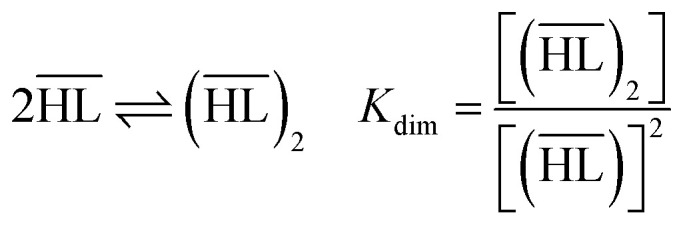
6
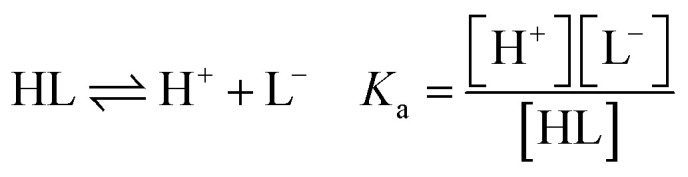
7
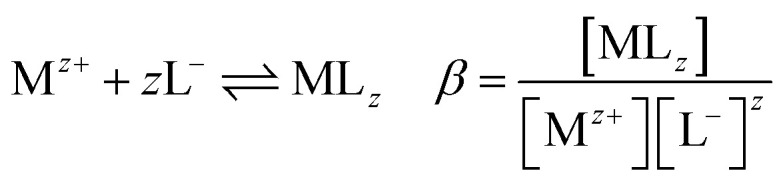
8
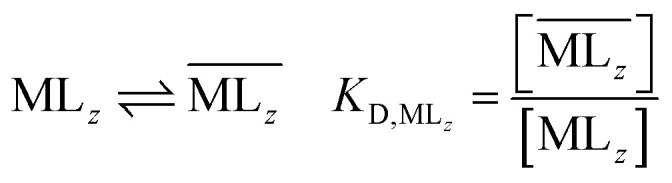
9
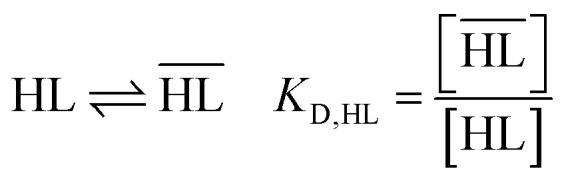


By combining eqn (4)–(9), the following expression of the extraction efficiency (in %) can be deduced:10
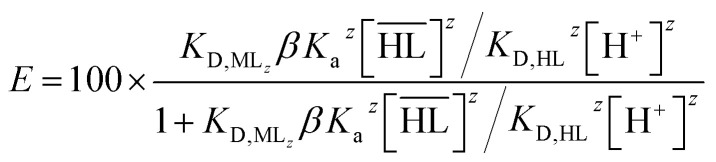


Thus, eqn (10) shows that the extraction efficiency at constant pH in the aqueous phase and at constant concentration of extractant in the organic phase can be improved provided that: (i) the metal–ligand complex is very stable in the organic phase (high value of *β*), (ii) the metal–ligand complex is highly soluble in the organic phase (high value of *K*_D_,_ML2_) and (iii) the p*K*_a_ of the cationic exchanger is as low as possible. Therefore, the steric hindrance, the complexing power, and the hydrophobicity of the extractants as well as the dipolar moment and the dielectric constant of the diluent can affect the values of these constants, and therefore, the extraction properties of the extraction solvent.

The influence of the diluents on copper(ii) recovery by 20% (wt) Acorga® M5640 was not reported in Fig. 1 as no significant effect was observed. At pH 0.6, the copper(ii) extraction efficiency reached 80% and nearly 100% at pH 1.5 for all diluents. Likewise, the nature of the diluent did not influence lithium extraction by DEHPA or Cyanex® 272 as shown in Fig. 1a and e. Conversely, a significant effect of the diluent on the extraction efficiency of cobalt(ii), nickel(ii) and manganese(ii) was observed when Cyanex® 272 and DEHPA were used as extractants.

The extraction efficiency curve of cobalt(ii) by DEHPA is shifted towards higher pH when EXILORE 180 or kerosene is used as diluent instead of the other ones. Regarding the influence of the diluent for the extraction of nickel(ii) by DEHPA, the extraction curves are located in the same region (pH_1/2_ = 4.2–4.4) when the diluent is kerosene EXILORE180, EXILORE230, ISANE IP175, DEV2138, DEV2139 or DEV1763. Conversely, the curves are shifted toward higher pH when these diluents are replaced by EXILORE205 or DEV2160. However, the most important effect of the diluent is observed with DEV2160 as the pH of half extraction reaches 5.5.

The behavior of manganese(ii) is quite different since the extraction curves are progressively shifted toward higher pH as follows: kerosene ≈ IsaneIP75 < EXILORE180 < (EXILORE230 ≈ EXILORE205) < (DEV2138 ≈ DEV2139 ≈ DEV2063) < (DEV1763 ≈ DEV2160 ≈ DEV2161).

Likewise, the diluent influences the extraction efficiency of cobalt(ii), nickel(ii) and manganese(ii) when Cyanex® 272 is used as extractant. The extraction efficiencies by Cyanex® 272 are shifted toward higher pH values when DEV 2261 is used instead of the other diluents for cobalt(ii) extraction and when DEV 2138 is replaced by the other diluents for nickel(ii) and manganese(ii) extraction.

**Fig. 2:**
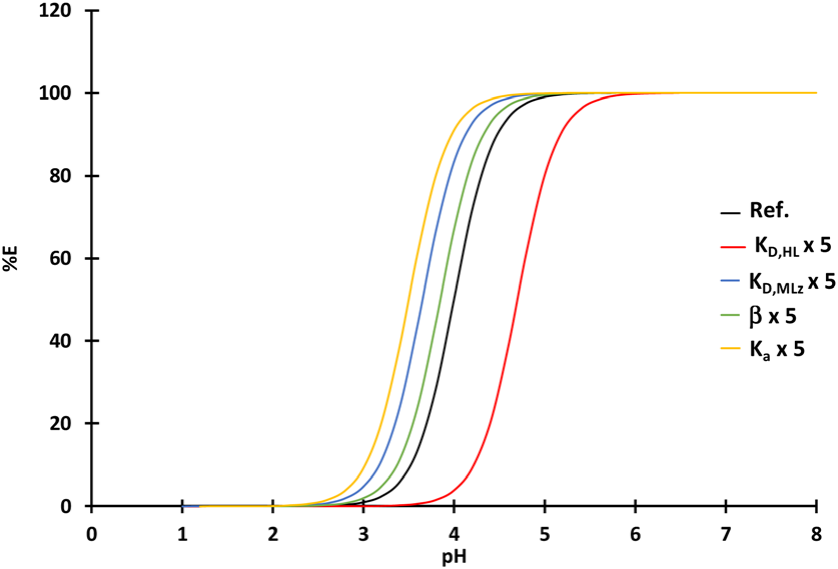
Influence of the acidity constant (*K*_a_), the complexation constant (*β*), the distribution constant of HL (*K*_D,HL_) and the distribution constant of ML_*z*_ (*K*_D,ML_*z*__) on the extraction efficiency calculated by eqn (10). Thermodynamic constants for the curve quoted “Ref.”: *K*_D,HL_ = 10^5^, *K*_D,ML_*z*__ = 10^5^, *β* = 10^5^ and *K*_a_ = 10^−4^. For the other curves these values are the same except one constant which has been multiplied by 5 to observe their influence on the curve.

Fig. 2 displays the influence of the acidity constant (*K*_a_), the complexation constant (*β*), the distribution constant of HL (*K*_D,HL_) and the distribution constant of ML_*z*_ between an aqueous phase and the extraction solvent (*K*_D,ML_*z*__) on the extraction efficiency (curves plotted by using eqn (10)). An increase of *K*_D,HL_ is responsible for a shift of the extraction curve toward higher pH whereas an increase of the other constants (*K*_a_, *β*, *K*_D,ML_*z*__) is responsible for a shift toward the lower pH values. Therefore, the shift of the extraction curves towards higher pH observed previously when the diluents are changed may result from an increase of the distribution constant of HL, *i.e.* an increase of the solubility of the extractant into the organic phase due to an increase of the diluent–extractant interactions. The extent of the shift can be mitigated by the increase of the other constants which are responsible for shifts towards the lower pH values.

### 3.2. Influence of diluents on the selectivity

**Fig. 3:**
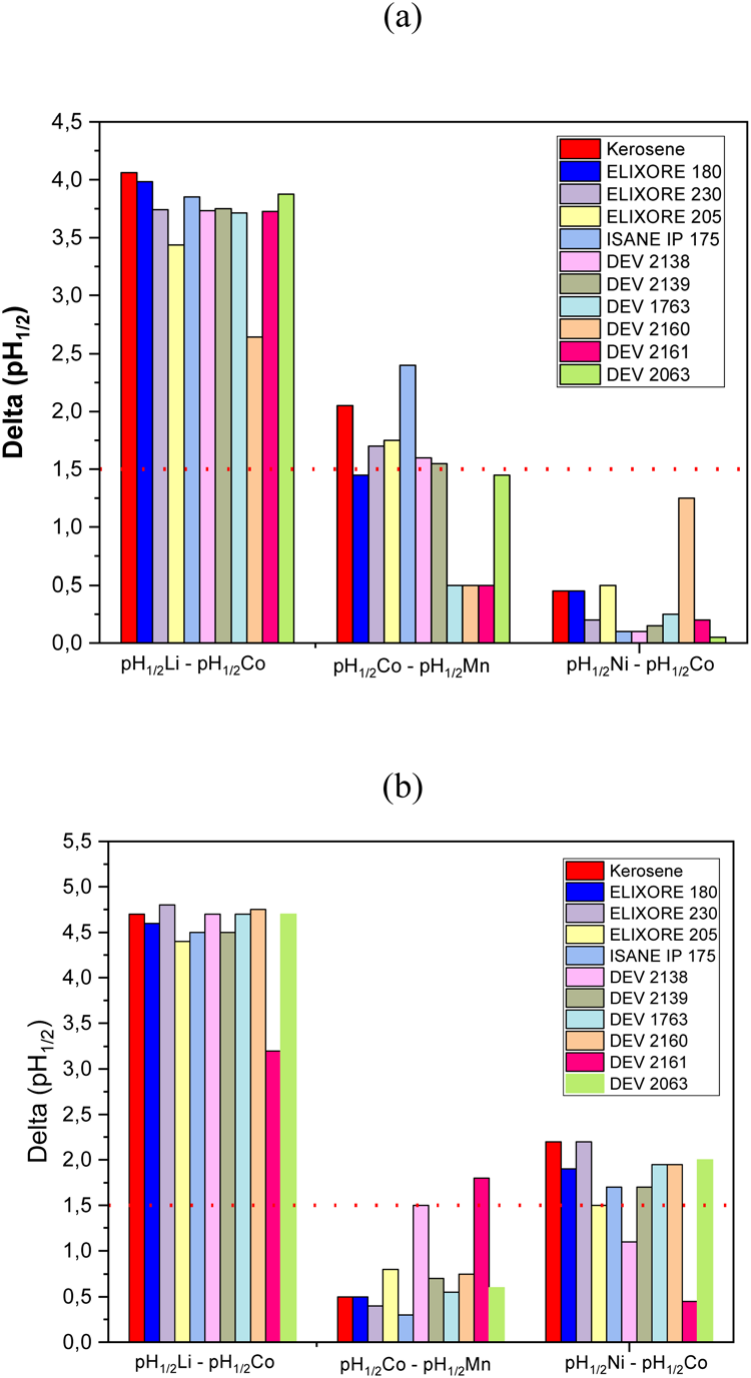
Differences in pH of half-extraction between lithium(i) and cobalt(ii) (pH_1/2,Li_–pH_1/2,Co_), cobalt(ii) and manganese(ii) (pH_1/2,Co_–pH_1/2,Mn_), and nickel(ii) and cobalt(ii) (pH_1/2,Ni_–pH_1/2,Co_) when the extraction solvent is composed of (a) 0.8 mol per L DEHPA and (b) 1 mol per L Cyanex 272 solubilized in diluents reported in Table 2 (O/A = 1, *T* = 25 °C, extraction time = 15 min).

The choice of the extraction system will rely on both the physicochemical properties (flash point, viscosity), the extraction properties (extraction efficiency, selectivity) and the environmental impact (evaluated by LCA, *vide infra*). The selectivity of an extraction solvent for M_1_ toward M_2_ can be assayed by calculating the difference in pH of half-extraction between M_1_ and M_2_ (pH_1/2,M_1__–pH_1/2,M_2__). Usually, it is inferred that a good separation can be achieved providing that pH_1/2,M_1__–pH_1/2,M_2__ ≳ 1.5. Fig. 3 shows the difference in pH of half-extraction between lithium(i) and cobalt(ii), cobalt(ii) and manganese(ii) as well as nickel(ii) and cobalt(ii) when the extraction solvent is composed of 0.8 mol per L Cyanex® 272 and 1 mol per L DEHPA diluted in the diluents reported in Table 3.

Fig. 3 shows that pH_1/2,Co_–pH_1/2,Mn_ decreased depending on the diluent as follows when DEHPA was used as extractant:

IsaneIP175 > kerosene ≫ Exilore205 ≈ Elixore230 ≈ DEV2138 ≈ DEV2161 ≈ EXILORE180 ≈ DEV2063.

The order was completely different when Cyanex® 272 was used instead of DEHPA (Fig. 3a):

DEV2161 > DEV2138 ≫ Exilore205 ≈ DEV2161 ≈ DEV2160 ≈ DEV2063 ≈ DEV1763 > kerosene ≈ EXILORE180 ≈ EXILORE230 > ISANEIP175.

Therefore, Co–Mn separation is much better with biosourced diluents (pH_1/2,Co_–pH_1/2,Mn_ = 1.8 and 1.5 for DEV2161 and DEV2138, respectively) when Cyanex® 272 is used as extractant whereas fossil diluents (pH_1/2,Co_–pH_1/2,Mn_ = 2.4 and 2.0 for Isane IP175 and kerosene, respectively) is better if the extraction solvent contains DEHPA. As expected, cobalt(ii)–manganese(ii) separation is better with DEHPA than Cyanex® 272.

Cobalt(ii)–nickel(ii) separation is not possible by using DEHPA as the difference of pH of half-extraction is lower than 1.5 whatever the diluent (Fig. 3b). When DEHPA was used as extractant, the values of pH_1/2,Ni_–pH_1/2,Co_ decreased as follows for the investigated diluents:

Kerosene = Exilore230 > DEV2063 ≈ DEV1763 ≈ DEV2160 ≈ Exilore180 > Exilore230 ≈ IsaneIP175 ≈ Exilore205 > DEV238 > DEV2161.

Almost all diluents led to relevant values of pH_1/2,Ni_–pH_1/2,Co_ event if the highest values were obtained for fossil diluents such as kerosene and Exilore230, *i.e.* pH_1/2,Ni_–pH_1/2,Co_ = 2.2. However, biosourced diluents such as DEV1763 and DEV2160 led to interesting values of pH of half-extraction (pH_1/2,Ni_–pH_1/2,Co_ = 2.0).

Therefore, the best biosourced diluent for cobalt(ii)–manganese(ii) separation with Cyanex® 272 by taking into account the extraction properties (pH_1/2,Co_–pH_1/2,Mn_ = 1.5), the flash point (64 °C) and the viscosity (*υ* = 1.17 mm^2^ s^−1^) is DEV2138. Regarding the extraction properties, the best biosourced diluent for nickel(ii)–cobalt(ii) separation are DEV2063, DEV2160 or DEV1763 as pH_1/2,Ni_–pH_1/2,Co_ = 2 in these diluents. All of these diluents exhibit a low kinematic viscosity. DEV2160 exhibits the lowest kinematic viscosity (*υ* = 1.07 mm^2^ s^−1^) but also the lowest flash point (FP = 49 °C). Finally, DEV1763 seems to be the best diluent as the flash point is very high (FP = 115 °C), the kinematic viscosity is high but comparable to EXILORE230 (*υ* = 2.4 mm^2^ s^−1^) and the difference in pH of half-extraction is high when Cyanex® 272 is used as extractant. DEV2160 is the only diluent with relevant extraction properties to separate cobalt(ii) and nickel(ii) when DEHPA is used as extractant even if the difference in pH of half-extraction is lower than with Cyanex® 272 (pH_1/2,Co_–pH_1/2,Mn_ = 1.25). However, as explained above, this diluent exhibits very low flash point.

### 3.3. CO_2_ footprint of the diluents

**Fig. 4:**
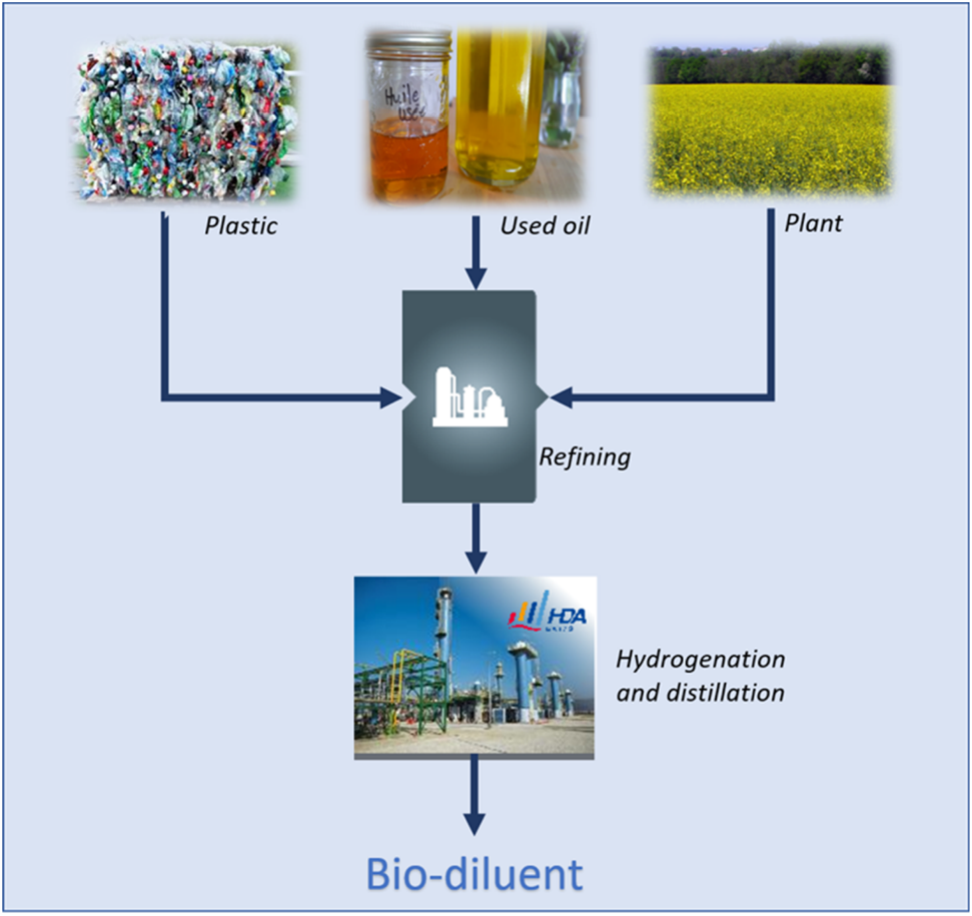
Manufacturing of biosourced diluents.

The process consists in refining the feedstocks, *i.e.* fossil, bio-oil (tall oil, vegetable oil, used cooking oil) or recycled plastics, and purifying the products from refining by performing hydrogenation and distillation as shown in Fig. 4.

For diluent coming from tall oil the process steps are: (i) conversion of wood into pulp mill, (ii) conversion of pulp mill into crude tall oil, (iii) distillation of crude tall oil to produce distilled tall oil and tall oil fatty acid, (iv) hydroprocessing of distilled tall oil and tall oil fatty acid into diesel fuels including impurities removal and hydro-isomerisation/hydrocracking, and (v) purification of biodiesel fuels into diluent by high pressurized hydrogenation. For diluent coming from spent plastics, the different process steps are: (i) waste collection of plastics, (ii) thermal cracking of the plastics which converts plastic waste into a hydrocarbons liquid, (iii) hydro-refining to remove impurities (iv) distillation, and (v) purification by high pressure hydrogenation. For diluent coming from used cooking oil, the different steps of the process are: (i) collection of used cooking oil, (ii) refining step including hydrocracking, isomerization and hydrogenation of used cooking oil to transform triglyceride into hydrocarbon liquid and (iv) purification by high pressure hydrogenation. ELIXORES are produced by high-pressure hydrogenation and distillation of diesel or kerosene feedstocks coming from refineries whereas kerosene is produced by a conventional refining process.

The LCA (cradle-to-gate) of the different routes described previously have been performed by using the EcoInvent 3.8 database. Each step contributes to the greenhouse gas emissions as shown in Table 3. It is important to note that the CO_2_ emissions are calculated per category of diluent (ELIXORE, kerosene, recycled plastic, *etc.*). This means that there is no difference in terms of CO_2_ emissions for the different diluents in the same category. Total emissions were calculated by summing the emissions from each process step, taking into account the inventories.

According to the ISO 14040/14044 and ISO 14067 standards, the greenhouse gas emissions of these feedstocks are 0 for recycled plastic, tall oil and used cooking oil wastes. The production of kerosene consists of two steps: oil production and refining. These two steps represent a CO_2_ emission of +462 kg CO_2_eq per t. ELIXORE diluents, based on diesel feedstock, generate more CO_2_ than kerosene because of the further refining and purification at the Oudalle plant.

The results presented in Table 4 show that the main stage that contribute to emits CO_2_ is the refining stage. In the case of bio-sourced diluents, the calculation of the global CO_2_ takes into account the CO_2_ credit. Indeed, during the growth of the plant, CO_2_ has been removed from the air and fixed in the biomass. After transformation, this captured CO_2_ is still in the product. Therefore, the percentage of carbon in the product was used to calculate the amount of CO_2_ which has been captured (negative value). In case of recycled plastic based diluent, the calculation of the global CO_2_ takes into account the avoided emission due to a usual end life of plastic, *i.e.* incineration.

**Table 4:** CO_2_ emissions (in kg CO_2_eq t^−1^) related to the different steps implemented for production of diluents including biosourced diluents^a^

	Process emissions	CO_2_ reduction	**GES total**
**Tall oil** (DEV2063)	+1413	−3120*	**−1707**
**Recycled plastic** (DEV2160, DEV2161)	+1495	−3700**	**−2205**
**Used cooking oil** (DEV2138, DEV2139)	+525	−3120*	**−2438**
**ELIXORES** (ELIXORE180, ELIXORE230, ELIXORE205)	+635	0	**+635**
**Kerosene**	+462	0	**+462**

a * means biogenetic CO_2_ credit and ** means avoided CO_2_ generated by incineration.

As a conclusion, the different diluents can be classified according to their carbon footprints as follow: ELIXORES (ELIXORE180, ELIXORE230, ELIXORE205) > kerosene > DEV2063 (tall oil) > DEV2160 and DEV2161 (recycled plastics) > DEV 2138 and DEV 2139 (used cooking oil).

## 4. Conclusion

**Fig. 5:**
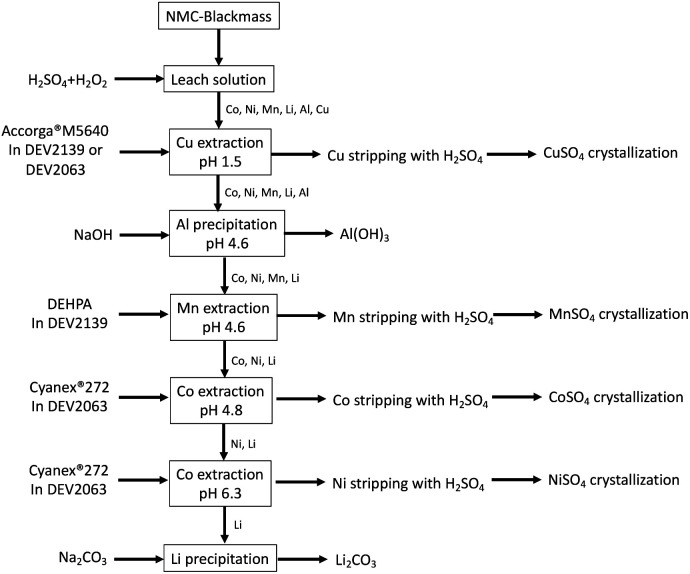
Low-carbon footprint solvent extraction flowsheet for the recovery of metals from spent lithium-ion batteries.

The present study shows that the use of low-carbon footprint diluents in solvent extraction for lithium-ion battery recycling is a good alternative to fossil kerosene. In particular, the diluents DEV2139 and DEV2063 produced from used cooking oil and tall oil, respectively, are interesting as they contain very low concentration of aromatics, their flashpoints are high, their viscosity is moderate and they can be advantageously used with DEHPA and Cyanex® 272 to extract selectively manganese(ii), and separate nickel(ii) and cobalt(ii). From the results of the present work, the flowsheet reported in Fig. 5 could be used to implement low-carbon footprint solvent extraction process for the recovery of copper, manganese, nickel, cobalt and lithium from spent lithium-ion batteries.

Fig. 5 shows a typical hydrometallurgical solvent extraction flowsheet that could be implemented to recover metals from spent lithium-ion batteries with low-carbon footprint diluents. After leaching the NMC-blackmass by sulfuric acid in the presence of hydrogen peroxide, 20% (wt) Acorga® M5640 diluted in DEV2139 or DEV2063 could be used to extract copper at pH 1.5 from the leach solution without significant co-extraction of the other metals. The pH of the resulting solution containing aluminum, cobalt, nickel, manganese and lithium could be adjusted to pH 4.6 with sodium hydroxide to precipitate aluminum. After solid/liquid separation, manganese could be extracted by using 0.8 mol per L DEHPA diluted in DEV2139 at pH 4.6. Under these conditions, losses of lithium, nickel and cobalt will be reduced. Afterwards, cobalt could be extracted selectively toward nickel by using 1 mol per L Cyanex® 272 diluted in DEV2063 at pH 4.8 and nickel could be afterwards selectively extracted towards lithium by using 1 mol per L Cyanex® 272 diluted in DEV2063 at pH 6.3 or by selective precipitation at pH 8. The resulting solution contains only lithium, which can be precipitated with sodium carbonate as lithium carbonate.

## Author contributions

Conceptualization: AC; formal analysis: AMA, AC; funding acquisition: AC; investigation: AMA, AC; project administration: AC; supervision: AC; visualization: AMA, AC; writing-original draft: AMA, AC; writing-review and editing: AC, BS, ZA, JYL.

## Conflicts of interest

There are no conflicts to declare.

## Supplementary Material

RA-013-D3RA04679F-s001
