# Supplementary material for: Low-carbon footprint diluents in solvent extraction for lithium-ion battery recycling
Source: RSC Adv. 2023 Aug 2;13(33):23334–45. doi: 10.1039/d3ra04679f (PMC10395664; doi:10.1039/d3ra04679f)
Supplement: RA-013-D3RA04679F-s001 [file RA-013-D3RA04679F-s001.pdf]

Electronic Supplementary Material (ESI) for RSC Advances.
This journal is © The Royal Society of Chemistry 2023

Low-carbon footprint diluents in solvent extraction for lithium-ion battery recycling

Aboudaye M. Ahamed[a], Benjamin Swoboda[b], Zubin Arora[b] , Jean Yves Lansot[b] and Alexandre Chagnes[a]

Supporting Information

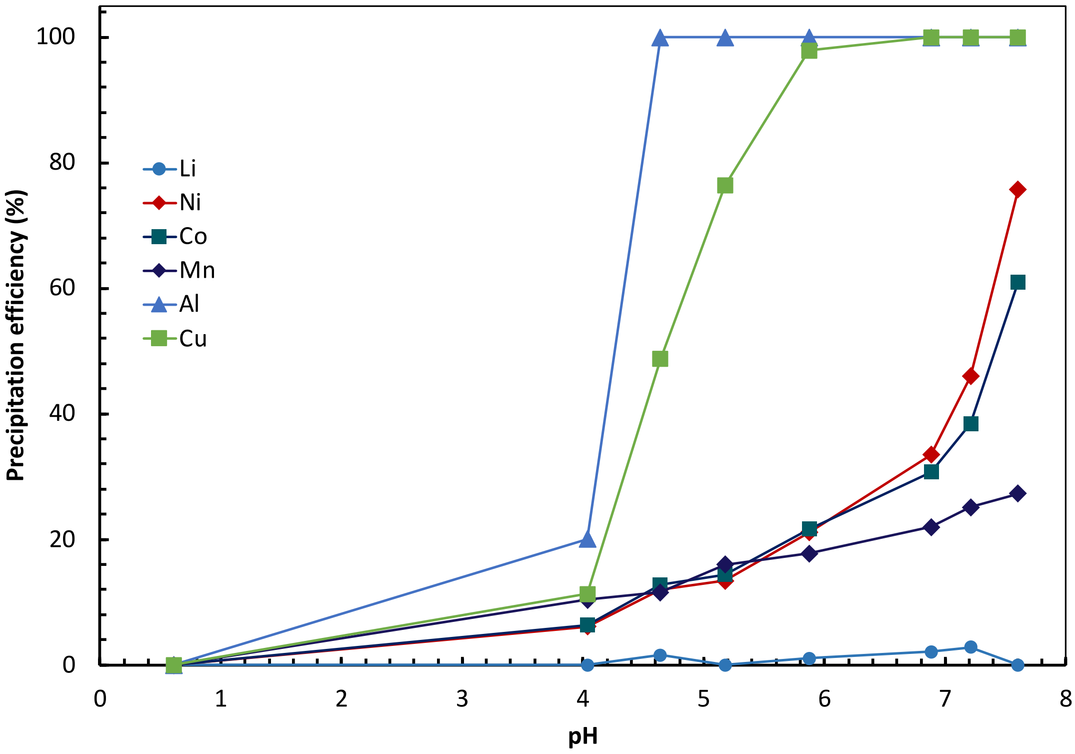

Figure S1: Precipitation curves as a function of pH.

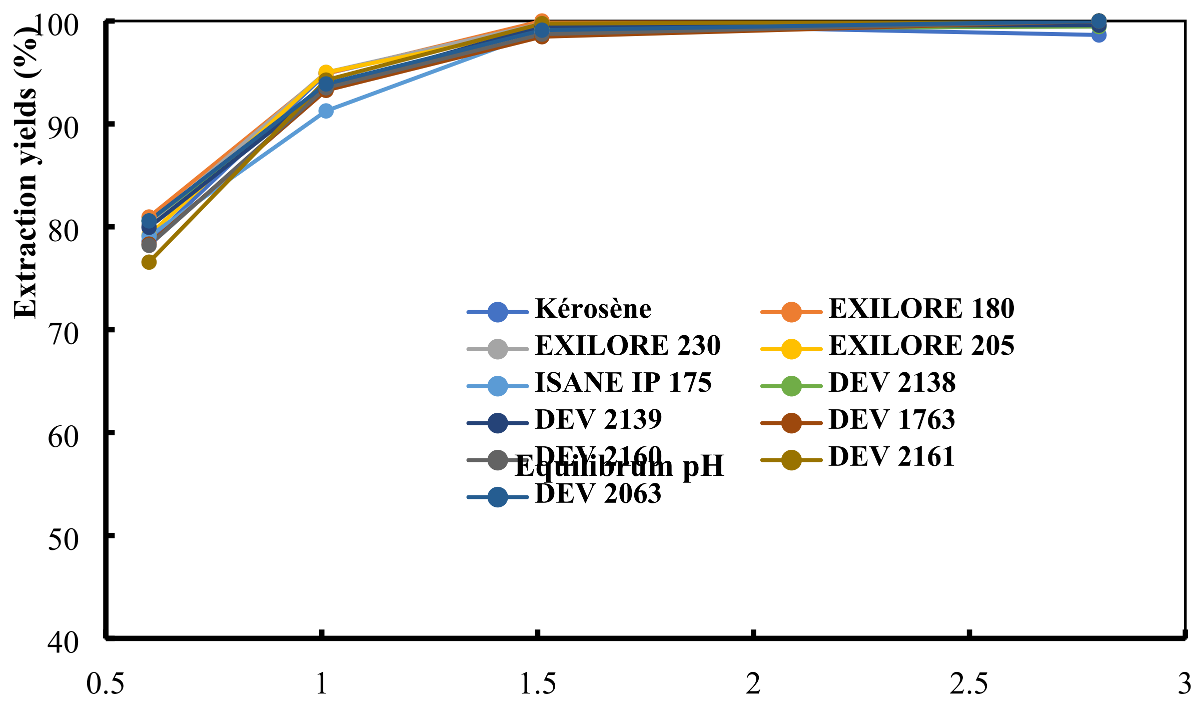

Figure S2: Extraction of copper by using Accorga M5640.
